# Burnout in the neonatal intensive care unit and its relation to healthcare-associated infections

**DOI:** 10.1038/jp.2016.211

**Published:** 2016-11-17

**Authors:** D S Tawfik, J B Sexton, P Kan, P J Sharek, C C Nisbet, J Rigdon, H C Lee, J Profit

**Affiliations:** 1 Division of Pediatric Critical Care, Department of Pediatrics, Stanford University School of Medicine and Lucile Packard Children's Hospital, Palo Alto, CA, USA; 2 Department of Psychiatry, Duke University School of Medicine, Duke University Health System, Durham, NC, USA; 3 Duke Patient Safety Center, Duke University Health System, Durham, NC, USA; 4 Perinatal Epidemiology and Health Outcomes Research Unit, Division of Neonatology, Department of Pediatrics, Stanford University School of Medicine and Lucile Packard Children's Hospital, Palo Alto, CA, USA; 5 California Perinatal Quality Care Collaborative; Palo Alto, CA, USA; 6 Center for Quality and Clinical Effectiveness, Lucile Packard Children's Hospital, Palo Alto, CA, USA; 7 Division of Pediatric Hospitalist Medicine, Department of Pediatrics, Stanford University, Palo Alto, CA, USA; 8 Quantitative Sciences Unit, Department of Medicine, Stanford University School of Medicine, Palo Alto, CA, USA

## Abstract

**Objective:**

To examine burnout prevalence among California neonatal intensive care units (NICUs) and to test the relation between burnout and healthcare-associated infection (HAI) rates in very low birth weight (VLBW) neonates.

**Study Design:**

Retrospective observational study of provider perceptions of burnout from 2073 nurse practitioners, physicians, registered nurses and respiratory therapists, using a validated four-item questionnaire based on the Maslach Burnout Inventory. The relation between burnout and HAI rates among VLBW (<1500 g) neonates from each NICU was evaluated using multi-level logistic regression analysis with patient-level factors as fixed effects.

**Results:**

We found variable prevalence of burnout across the NICUs surveyed (mean 25.2±10.1%). Healthcare-associated infection rates were 8.3±5.1% during the study period. Highest burnout prevalence was found among nurses, nurse practitioners and respiratory therapists (non-physicians, 28±11% vs 17±19% physicians), day shift workers (30±3% vs 25±4% night shift) and workers with 5 or more years of service (29±2% vs 16±6% in fewer than 3 years group). Overall burnout rates showed no correlation with risk-adjusted rates of HAIs (*r*=−0.133). Item-level analysis showed positive association between HAIs and perceptions of working too hard (odds ratio 1.15, 95% confidence interval 1.04–1.28). Sensitivity analysis of high-volume NICUs suggested a moderate correlation between burnout prevalence and HAIs (*r*=0.34).

**Conclusion:**

Burnout is most prevalent among non-physicians, daytime workers and experienced workers. Perceptions of working too hard associate with increased HAIs in this cohort of VLBW infants, but overall burnout prevalence is not predictive.

## Introduction

In 1999, the Institute of Medicine estimated that medical errors are responsible for up to 98 000 deaths annually in the United States of America.^1^ Recent studies suggest that this may have been an underestimate, with the true number of premature deaths related to preventable harm estimated up to 440 000, or approximately one-sixth of the deaths in the country each year.^2, 3^ Critically ill patients often require fast-paced, complex and precise care, resulting in an increased propensity toward errors along with increased vulnerability. Very low birth weight (VLBW) infants are particularly vulnerable to errors, and adverse events occur with up to 10-fold variation among neonatal intensive care units (NICUs).^4^

Neonatal infections are especially hazardous, with sequelae including prolonged length of stay,^5^ adverse neurodevelopmental outcomes^6^ and increased mortality.^5, 7, 8^ Higher infection rates have been linked to poor performance in other areas of safety culture including teamwork and safety climate,^9, 10^ raising concern for burnout as a source of decreased quality of healthcare,^11, 12^ particularly in relation to critically ill pediatric patients.

Burnout describes a condition of fatigue, detachment and cynicism resulting from prolonged high levels of stress.^13^ In the critical care setting, burnout rates may be driven by high workload, frequent changes in technology and guidelines, endeavors for high-quality care and emotional challenges of dealing with critically ill patients and their families.^14, 15, 16, 17^ Burnout affects 27–86% of healthcare workers,^18, 19, 20^ with over half of physicians reporting burnout^21^ and around one-third of nurses and physicians meeting criteria for severe burnout.^18, 22^

Levels of burnout among different types of NICU providers are poorly characterized, and the effect of burnout on neonatal quality of care is unknown. The objectives of this study were to:

1. Describe variation of NICU caregiver burnout by provider characteristics.
2. Analyze the relation between caregiver burnout and healthcare-associated infection (HAI) rates among VLBW (<1500 gm) infants.

## Materials and methods

This cross-sectional study links survey data to subsequent clinical outcomes data derived from a population-based clinical registry among 44 California NICUs.

### Sample and procedure

#### Selection of NICUs

The California Perinatal Quality Care Collaborative (CPQCC) is a multi-stakeholder group of public and private neonatal healthcare providers, including over 130 member hospitals and accounting for the majority of preterm infants requiring critical care in the state of California. This cross-sectional survey was performed among a voluntary sample of CPQCC NICUs participating in a Delivery Room Management Quality Improvement Collaborative.^23^ For the current study, we offered to analyze and provide feedback on a survey of safety culture and workforce engagement to all 61 NICUs who participated in the improvement initiative, 44 of which accepted. The survey was administered at the beginning of the improvement initiative, between June and September 2011.

Staff members with a 0.5 full-time equivalent or greater time commitment to the NICU for at least the four consecutive weeks before survey administration were eligible for inclusion. Paper-based surveys were administered during routine departmental and staff meetings. Respondents returned surveys to a locked box or sealable envelope to maintain confidentiality. Individuals not present in routine meetings were hand-delivered a survey, pencil and return envelope. This administration technique has generated high response rates^24, 25^ comparable to other studies of similar methodology.^26^ CPQCC staff administered the survey and transmitted a de-identified data set to Drs. Profit and Tawfik for analysis. Sensitivity analysis was performed using the subset of NICUs with a >50% response rate.

#### Selection of patients

To capture outcomes subsequent to survey responses, clinical data routinely submitted to the CPQCC by Collaborative members reflecting VLBW infants born between 1 January 2012 and 31 December 2013 were linked to the survey data using unique identifiers for NICUs and patients. Similar to a previous approach used by our group to assess the quality of care delivery in the NICU setting,^27, 28, 29^ we excluded infants with severe congenital anomalies to reduce systematic bias in infection rates between NICUs that are the result of the need for prolonged parenteral nutrition and surgical intervention. We used multiyear analysis due to the small number of VLBW infants cared for in some institutions. Sensitivity analysis was performed using the subset of NICUs with 5 or more predicted HAIs during the study period, corresponding to NICUs with 60 or more VLBW infants.

### Measures

#### Survey data

Measures relevant for this study were part of a larger survey on safety culture and organizational determinants of quality. The four-question emotional exhaustion subset of the Maslach Burnout Inventory^30^ was used, which has shown to be reliable and valid in other settings.^31^ The psychometric properties of the emotional exhaustion subset have performed favorably in recent analysis and suggested its appropriateness as a marker of overall burnout climate.^13^

A burnout score for each respondent was computed by taking the mean of the four items and transforming it to a 0–100 point scale using the following formula:

Burnout score for a respondent=((mean of the burnout items)−1) × 25

To calculate the percent of respondents who are burned out (that is, percent that agree with burnout items), one calculates the percent of respondents who received a scale score of 50 or higher. This 50% threshold groups ‘neutral' responses together with ‘agree' responses as previously described in the literature.^13, 25^ This metric has been found to be meaningful to providers when used in safety culture assessments.^13, 32^

We assessed provider perception of nurse staffing adequacy by responses to the following questions from the Safety Attitudes Questionnaire:^25^ ‘I have the support I need from others in this NICU to care for patients' and ‘The staffing levels in this NICU are sufficient to handle the number of patients'.

The survey also captured respondent characteristics including job position, years in specialty, primary work area (pediatric, adult or both), gender and predominant work shift. Job positions included attending physicians, fellow physicians, neonatal nurse practitioners, registered nurse, respiratory care practitioners and others.

#### Clinical data

CPQCC clinical data are equivalent in their definitions to those of the Vermont Oxford Network,^33^ and undergo a series of quality checks to ensure completeness and accuracy. Healthcare-associated infection rates for each NICU were calculated using standard CPQCC definitions of this measure (also called ‘any nosocomial infection'), which includes any bacterial or fungal infection acquired after 3 days of age. For infants that were transferred to another facility, attribution of infection was defined to include those acquired ‘here' and ‘here and elsewhere'. We adjusted infection rates according to a severity of illness model we developed in a previous study.^29^ The variables included gender, gestational age at birth, 5 min Apgar score, small for gestational age (<10th percentile) and birth at the NICU under investigation or outborn.

#### Data analyses

Descriptive statistics including frequencies, means and s.d.'s were used to describe survey responses and respondent demographics. Burnout prevalence was calculated as described above, resulting in a proportion of total respondents per NICU reporting symptoms of burnout, powered to detect a 10% difference in burnout. For job position analyses, we pooled respondents into ‘physician' (attending and fellow) and ‘non-physician' (nurse practitioner, registered nurse, respiratory therapist and other) groups due to the small number of respondents from some of the categories.^13, 32^

Basic descriptive statistics examined the variation in HAI rates across NICUs, and were correlated to burnout prevalence using Pearson's correlation coefficients. Hierarchical modeling was employed to account for infant-level clinical risk factors as well as respondent characteristics nested within NICUs.^29^

All statistical analyses were performed using SAS version 9.4. The study was approved by the Institutional Review Board at Stanford University with waiver of informed consent.

## Results

### Objective 1 – describe the variation of NICU caregiver burnout by provider type.

Forty-four NICUs participated in this study, with 2073 of 3294 surveys returned for a 62.9% response rate. Individual NICU response rates averaged 69.7% (s.d. 19.8%, range 21.7–100%). Table 1 lists respondent characteristics, which indicated 59.9% of respondents with 11 or more years in their specialty, and 2.4% of respondents with <1 year of experience. Day shift was the most common (47.9%), with night shift (32.2%) and variable shift (15.7%) accounting for the majority of the others.

**Figure 1:**
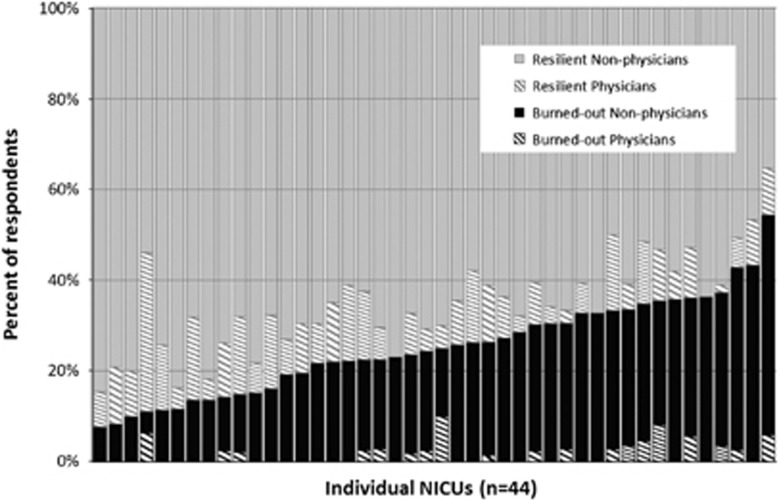
Burnout distribution in 44 NICUs, *n*=2073. Burnout was variable among NICUs (*F*=2.86, *P*<0.001) and physicians reported lower burnout prevalence than non-physicians (17±19% vs 28±11%, *P*<0.001). Physicians in textured bars.

**Figure 2:**
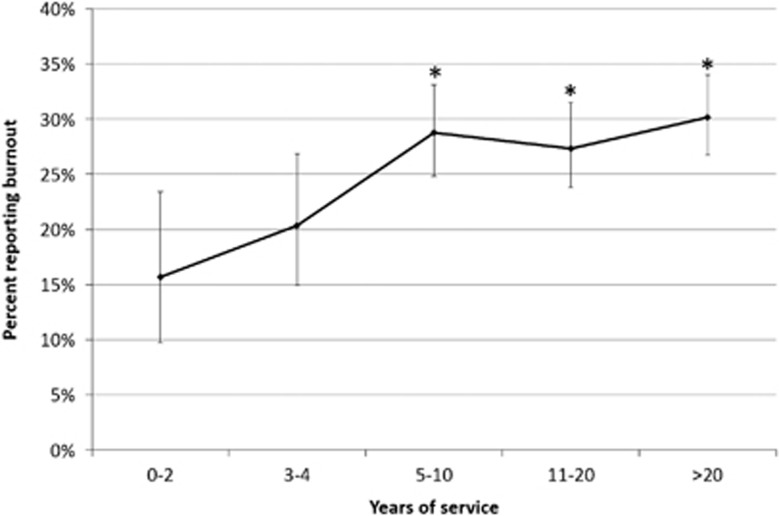
Burnout by years of service. *n*=2073 respondents in 44 NICUs, with 95% CIs. Providers with fewer than 3 years experience reported the lowest burnout (16±6% vs 30±4% in >20 years group, *P*=0.001). *denotes statistical significance (*P*<0.05) from the fewer than 3 years group.

One quarter (25.2%±10.1) of respondents reported symptoms consistent with burnout, and individual NICU burnout prevalence ranged from 7.5 to 54.4%. Across NICUs, non-physicians reported higher rates of burnout than physicians (28±11% vs 17±19%, *P*<0.001), as shown in Figure 1. Respondents with 5 or more years of experience reported higher burnout prevalence than those with fewer than 3 years (29±2% vs 16±6%, *P*=0.002) as shown in Figure 2.

### Objective 2 – analyze the relation between caregiver burnout and HAI rates.

Table 1 shows the characteristics of the clinical sample. Individual NICUs cared for 16 to 399 infants during the study period. Of the 4386 VLBW infants included in the study, 366 (8.3%) experienced an infection during the study period. Seventy-four percent of the infants were cared for at their birth hospital. The risk-adjusted mean percentage of HAI was 8.1% with a range of 0–25.4%. Overall burnout prevalence did not show correlations with HAI rates among the complete cohort of NICUs (*r*=−0.133, *P*=0.40).

Table 2 shows patient level associations of burnout items and the burnout scale after adjustment for clinical characteristics. All parameter estimates except feelings of frustration pointed in the expected direction of lower HAI rates with fewer burnout symptoms. Items 1 through 3 and the composite burnout score were not independently associated with HAI rates, although item 1 (‘I feel fatigued when I get up in the morning and have to face another day on the job' *P*=0.09) showed a trend towards significance. Item 4 (‘I feel I am working too hard on my job' *P*=0.01) was significantly and independently associated with increased HAI rates, such that the odds of an infant contracting a HAI was 11.5% lower with each 10% decrease in NICU respondents reporting this burnout symptom. In the safety culture literature, a 10% change has been regarded as a significant improvement.^34^

Perception of staffing adequacy was negatively associated with burnout prevalence, with results consistent among those reporting sufficient support from others to take care of patients (*r*=−0.51, *P*<0.001) and sufficient staffing levels for the number of patients (*r*=−0.47, *P*=0.001).

**Figure 3:**
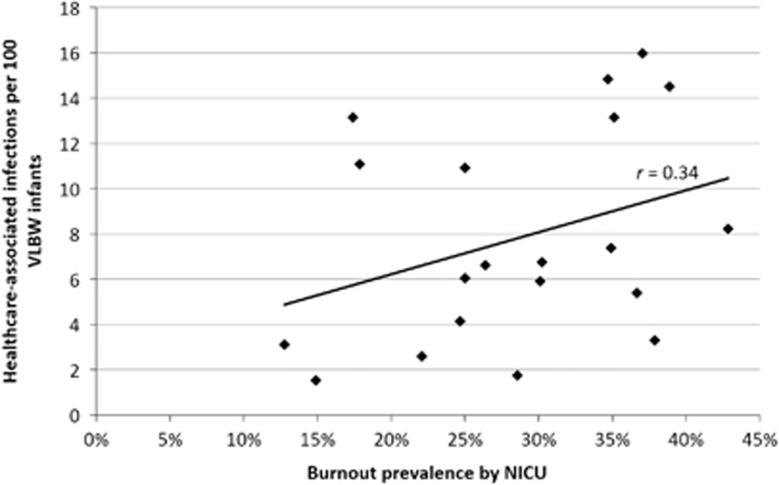
Relation between burnout prevalence and adjusted HAI rates, limited to NICUs with 60 or more VLBW infants and >50% survey response rate. *n*=1188 respondents in 20 NICUs. Positive Pearson's correlation between burnout and infections was noted, but this was not statistically significant (*r*=0.34, *P*=0.14).

Due to the potential for spurious results in the calculation of infection rates among small NICUs and burnout prevalence among those with low survey response rates, Figure 3 shows the subset of NICUs with greater than 60 VLBW infants (thus 5 or more predicted infections based on the overall HAI rate of 8.3% in this cohort) during the study period and a >50% survey response rate. This subset demonstrated positive but not statistically significant correlation between burnout prevalence and HAI (*r*=0.34, *P*=0.14). Patient level associations within this subset are shown in Table 2, which showed significant and independent associations between HAI rates and fatigue (*P*=0.008) as well as perceptions of working too hard (*P*=0.002). Additional sensitivity analysis expanded to include patients with severe congenital anomalies found no significant difference as compared with the index cohort.

## Discussion

The major finding of this study is that neonatal provider burnout prevalence is highest among non-physicians, day shift workers and more experienced workers. In addition, perceptions of working too hard are associated with increased HAIs.

Non-physicians reported higher burnout scores on average, a cohort composed primarily of nurses in this study. This is consistent with the lower perceptions of other safety culture components observed among nurses, including safety climate, teamwork and perceptions of management.^24, 32^ These differences may be due to personal characteristics, job-related factors or a combination of the two. Although fellow physicians reported the lowest burnout prevalence of any cohort, the small number of respondents in this category prevents firm conclusions regarding this subset. Their responses are consistent, however, with the observed trend of lower burnout among less experienced providers in general.

Providers with 5 or more years of experience reported higher rates of burnout than their less experienced peers. It is possible that these providers progressed to burnout as the result of the cumulative effect of repeated stressors. In addition, attrition of burned out providers may counterbalance continued progression of active providers toward burnout, resulting in the observed overall stable prevalence throughout the cohorts of experienced workers.

Providers working primarily day shift also reported higher rates of burnout. Although those with more experience are also more likely to work day shifts, this association was observed independently among workers with 3 or more years of experience. Although night shift work can be challenging in its disruption of circadian rhythm and difficulty coordinating responsibilities outside of work, it is possible that day shift work is more demanding for many due to diagnostics, treatment activities, difficult conversations with families and withdrawals of care being performed during the daytime hours whenever possible.

Overall burnout prevalence did not correlate to HAI rates in the complete cohort of NICUs. However, item-level associations with HAI were seen among those reporting perceptions of working too hard (*P*=0.01). We speculate that overworked providers may be less likely to follow institutional protocols that they perceive as unnecessary or overly burdensome. They also may be less likely to notice errors or omissions in healthcare delivery. The fear of retaliation or emotional expenditure of voicing concerns to other team members, hospital administration or family members may be insurmountable to a fatigued or overworked provider, instead rationalizing an approach of nonintervention when such errors are identified.^35^ Conversely, increased HAI rates may contribute to burnout by increasing patient acuity and lengths of stay, exacerbating staffing shortages and contributing to moral distress.^8, 36^

Nursing staffing shortages have been well-described in the adult literature as potential drivers for nurse burnout and decreased quality of care,^37, 38, 39^ with nursing shortages also correlated to increased neonatal infections.^40^ Clinical experience and our findings in this study suggest increased burnout in understaffed units with higher infection rates during times when nurses feel overworked, likely times when attention to detail necessary to prevent infections goes lacking. Therefore, we propose that managers increase sensitivity to episodes of staff shortages, with the use of ‘time outs' and daily huddles to raise awareness, encourage protocol adherence and peer support, and increase general vigilance.

Over 40% of the NICUs sampled had fewer than 5 predicted HAIs, making accurate assessment of the true HAI rate challenging, and 5 additional NICUs had fewer than 50% of eligible staff respond to the questionnaire, decreasing the ability to accurately determine burnout culture in those units. Sensitivity analysis using the data points least susceptible to these sampling errors showed a moderately strong correlation between burnout and HAI, and all item-level associations with HAI were stronger, most notably among those reporting symptoms of fatigue and perceptions of working too hard. The associations lacking in statistical significance may be limited by small sample size of the subgroup, and the strength of correlation merits further investigation on a larger scale.

This study must be interpreted in the context of its design. This cross-sectional study cannot determine causality of the observed associations, though our findings are in line with prior studies in adults.^11, 12^ The study is susceptible to response bias at both the NICU and individual respondent levels, as unmotivated NICUs and burned out individuals may have unpredictable propensities to participate. Overall, our response rate of nearly 63% compares favorably with other similar studies.^26^ Not all respondents worked exclusively in the NICU being evaluated, with likely additional unmeasured predictors and effects of burnout for these individuals. Item-level and subgroup associations resulted from *post hoc* analyses, and require replication in prospective studies. This study likely carries relevance for the population as a whole despite regional practice variation, as it represents a large neonatal burnout cohort stemming from a diverse sample of NICUs across the most populous state in the United States.

## Conclusion

We found prevalent but variable burnout among this cohort of NICUs, with non-physicians and more experienced providers reporting the highest burnout prevalence. Perception of working too hard was associated with increased HAI. NICUs with higher volumes showed a moderate correlation between burnout prevalence and HAI, and HAI rates associated most strongly with symptoms of fatigue and perceptions of working too hard. Interventions to prevent and reduce burnout among NICU providers may be important for reducing HAIs in these fragile patients.

## Figures and Tables

**Table 1:** Description of survey respondents and clinical sample. Sensitivity analysis describes NICUs with >60 VLBW infants and >50% survey response rate

	*Full cohort*	*Sensitivity analysis*
	N=*44*	N=*20*
*NICU level*, n *(%)*		
Size		
Intermediate	6 (13.6)	–
Community	28 (63.6)	14 (70)
Regional	10 (22.7)	6 (30)
		
Survey response rate		
<50%	10 (22.7)	–
50–75%	15 (34.1)	11 (55)
>75%	19 (43.2)	9 (45)
		
VLBW infants		
<60	19 (43.2)	–
60–155	17 (38.6)	14 (70)
>155	8 (18.2)	6 (30)

*Respondent level*, n *(%)*	N=*2073*	N=*1188*
Females	1697 (84.8)	980 (85.1)
		
Primarily		
Adult care provider	63 (3.6)	36 (3.6)
Pediatric care provider	1537 (88.3)	896 (89.2)
Both	140 (8.1)	72 (7.2)
		
Typical shift		
Days	894 (47.9)	515 (47.7)
Evenings	79 (4.2)	44 (4.1)
Nights	602 (32.2)	367 (34.0)
Variable	293 (15.7)	154 (14.3)
		
Position		
Attending physician	204 (10.0)	102 (8.8)
Fellow physician	31 (1.5)	12 (1.0)
Neonatal nurse practitioner	35 (1.7)	27 (2.3)
Registered nurse	1464 (71.7)	841 (72.1)
Respiratory therapist	286 (14.0)	171 (14.7)
Other	21 (1.0)	13 (1.1)
		
Years in specialty		
<6 months	20 (1.0)	15 (1.3)
6–11 months	27 (1.4)	15 (1.3)
1–2 years	74 (3.8)	35 (3.1)
3–4 years	192 (9.7)	116 (10.3)
5–10 years	476 (24.2)	307 (27.1)
11–20 years	538 (27.3)	317 (28.0)
21 years or more	643 (32.6)	326 (28.8)

*VLBW infants, mean (±s.d.) or* n *(%)*	N=*4386*	N=*3145*
Gestational age at birth (weeks)	28.3 (±2.9)	27.8 (±3.0)
Birth weight, g	1070 (±282)	1028 (±300)
Small for gestational age	842 (19.2%)	542 (17.2%)
Male sex	2186 (49.8%)	1546 (49.2%)
Five minute Apgar score		
<4	200 (4.6%)	289 (9.2%)
4–6	753 (17.2%)	548 (17.4%)
>6	3414 (78.2%)	2308 (73.4%)
Inborn	3260 (74.3%)	2542 (80.8%)
Healthcare-associated infection	366 (8.3%)	236 (8.4%)

Abbreviations: NICUs, neonatal intensive care units; VLBW, very low birth weight.

**Table 2:** Relation between HAI rates and burnout items

		*Full cohort*			*Sensitivity analysis*		
*Burnout item*	*Mean PPR (*s.d.)	*Parameter estimate (s.e.)*	P*- value*	*OR (95% CI)*	*Parameter estimate (s.e.)*	P*- value*	*OR (95% CI)*
I feel fatigued when I get up in the morning and have to face another day on the job.	36.6 (10.5)	1.26 (0.74)	0.09	1.13 (0.98–1.31)	**2.42** (0.92)	0.008	1.27 (1.06–1.52)
I feel burned out from my work.	30.9 (10.1)	0.24 (0.64)	0.71	1.02 (0.90–1.16)	0.91 (0.75)	0.22	1.10 (0.95–1.27)
I feel frustrated by my job.	36.4 (12.7)	−0.07 (0.46)	0.87	0.99 (0.91–1.09)	0.29 (0.49)	0.54	1.03 (0.94–1.13)
I feel I am working too hard on my job.	38.5 (11.6)	**1.43** (0.55)	0.01	1.15 (1.04–1.28)	**1.96** (0.64)	0.002	1.22 (1.07–1.38)
Composite burnout score >50	25.9 (10.8)	0.24 (0.63)	0.70	1.02 (0.91–1.16)	1.00 (076)	0.19	1.10 (0.95–1.28)

Abbreviations: 95% CI, 95% confidence interval; HAI, healthcare-associated infection; NICU, neonatal intensive care units; OR, odds ratio; PPR, percent positive rate; VLBW, very low birth weight. Full cohort: *n*=2073 respondents and 4386 infants in 44 NICUs. Sensitivity analysis: *n*=1188 respondents and 3145 infants in 20 NICUs, limited to NICUs with >60 VLBW infants and >50% survey response rate. Fixed model with adjustment for sex, small for gestational age, gestational age in weeks, outborn and Apgar score at 5 min. Analysis at the patient level. Bold font denotes statistically significant values (*P*<0.05).
